# The *NME7* Gene Is Involved in the Kinetics of Glucose Processing

**DOI:** 10.3390/ijms26199821

**Published:** 2025-10-09

**Authors:** Daniela Vejražková, Josef Včelák, Markéta Vaňková, Petra Lukášová, Michaela Svojtková, Tereza Grimmichová, Hana Kvasničková, Andrea Tura, Lucie Šedová, Ondřej Šeda, Kateřina Škultéty, Běla Bendlová

**Affiliations:** 1 Institute of Endocrinology, Narodni 8, 110 00 Prague, Czech Republic; jvcelak@endo.cz (J.V.); mvankova@endo.cz (M.V.); plukasova@endo.cz (P.L.); msvojtkova@endo.cz (M.S.); tgrimmichova@endo.cz (T.G.); hkvasnickova@endo.cz (H.K.); bbendlova@endo.cz (B.B.); 2 Institute of Neuroscience, National Research Council (CNR), Corso Stati Uniti 4, 35127 Padova, Italy; andrea.tura@cnr.it; 3 Institute of Biology and Medical Genetics, First Faculty of Medicine, Charles University and General University Hospital in Prague, Albertov 4, 128 00 Prague, Czech Republic; lucie.sedova@lf1.cuni.cz (L.Š.); ondrej.seda@lf1.cuni.cz (O.Š.); 4 Second Faculty of Medicine, Charles University, V Uvalu 84, 150 06 Prague, Czech Republic; skultety.katerina@seznam.cz

**Keywords:** glucose metabolism, type 2 diabetes mellitus, glycemic curve shape, glucose tolerance test, *NME7* gene, ciliopathies

## Abstract

Given that type 2 diabetes mellitus is common in several ciliopathies, the *NME7* gene (non-metastatic cells 7), encoding a recognized member of the ciliome, was studied in connection with glucose metabolism. The aim was to find out whether the variability in the gene is associated with the response to administered glucose during the 3 h oral glucose tolerance test. The study included 1262 individuals with different levels of glucose tolerance. Glycemic curves were categorized according to their shape as monophasic, biphasic, triphasic, and more complex multiphasic. The analysis showed a significant association of five linked *NME7* polymorphisms with the biphasic course of the glycemic curve, a shape that has been shown to be metabolically protective. Specifically, minor alleles of rs4656659 and rs2157597 in combination with wild-type alleles of rs10732287, rs4264046, and rs10800438 were more frequent within the biphasic category. Moreover, haplotype analysis confirmed higher insulin sensitivity in carriers of this specific haplotype. In conclusion, a cluster of five linked *NME7* polymorphisms showed an association with a biphasic glycemic curve. Considering the health benefits of the biphasic curve in terms of glycoregulation and taking into account the demonstrated link of the *NME7* haplotype with insulin sensitivity, variability in the *NME7* gene represents another piece of the complex mosaic influencing healthy energy processing.

## 1. Introduction

### 1.1. Factors Disrupting Glucose Homeostasis

Type 2 diabetes mellitus (T2DM) is a chronic metabolic disorder characterized by hyperglycemia in the context of insulin resistance (IR) and impaired insulin secretion. The manifestation of the disease is usually preceded by a long-lasting period of impaired fasting glucose (IFG) and/or impaired glucose tolerance (IGT). T2DM is associated with serious microvascular and macrovascular complications, including diabetic retinopathy, nephropathy, or vascular diseases threatening heart attack, stroke, or impaired blood circulation in the lower limbs, which may even result in the need for amputation. T2DM reduces life expectancy by approximately 10 years [1]. In the Czech Republic, the prevalence reaches almost 10%, with an alarming rise in younger age groups [2], probably in connection with the decrease in regular physical activity together with inadequate caloric intake resulting in increased prevalence of obesity. Excessive weight may also be a consequence of chronic stress or a result of long-term exposure to endocrine disruptors, which destroy the hormonal balance in the body. Prenatal developmental conditions may also play a role [3,4,5]. Diagnosis of overt T2DM thus reflects the interaction between a wide range of developmental and external factors with a genetic and epigenetic background [6].

Concerning genetic predisposition to the development of T2DM, genome-wide association studies and subsequent large-scale meta-analyses led to the identification of more than 600 genetic loci that modulate the risk of developing T2DM or are involved in closely related regulatory mechanisms, such as variations in fasting plasma glucose [7]. Currently, multi-omics analyses enhance the role of genotype data in developing new treatment strategies based on targeted therapy, advancing towards precision medicine tailored to the individual [8]. Our findings would like to contribute to the knowledge that underpins this approach.

### 1.2. Oral Glucose Tolerance Test Course

The oral glucose tolerance test (OGTT) and the derived indices of insulin sensitivity and insulin secretion are being used as the most suitable approach for assessing glucose metabolism in epidemiological studies [9].

The shape of the glycemic curve reflects the dynamics of the beta cell response to a glucose stimulus. The fasting blood glucose level before the start of the test and at the 120th minute after drinking a solution containing 75 g of glucose serve as diagnostic criteria for metabolic disorders like IFG, IGT, or T2DM. In the past years, many scientific teams have been engaged in the analysis of the shape of the glycemic curve, aiming to capture and interpret its physiologically relevant information [10,11,12,13]. The 2 h tests predominate, but studies evaluating the course of the 3 h OGTT are no longer rare. Blood glucose is usually measured at 30-minute intervals, and the curve shape is defined by the pattern of rising and subsequent falling glucose concentrations. The monophasic curve characterized by a single peak before the gradual decline has been associated with impaired glucose regulation and with the presence of other components of metabolic syndrome [11,12], whereas biphasic, triphasic, and multiphasic curves characterized by more complex trajectories with a greater number of inflections were shown to be more favorable for glucose homeostasis and other metabolic aspects [14].

### 1.3. The NME7 Gene

This gene, located on chromosome 1 (locus 1q24.2), encodes the NME7 protein (nucleoside diphosphate kinase 7, non-metastatic cells 7), an acknowledged member of the ciliome involved in the biogenesis and function of cilia. Variability in human motile cilia-related genes may represent a connection between cilia motility, islet beta cell function, and disturbances in glucose homeostasis, even T2DM. A deeper understanding of this functional interdependence may provide a new potential target for a personalized approach in diabetes treatment. Cilia have diverse roles in energy homeostasis, central signaling, and peripheral regulation of energy metabolism in adipose tissue and the pancreas. Mutations in genes encoding ciliary proteins result in signaling defects and lead to disorders such as obesity and T2DM [15]. Multiple human genetic diseases, such as polycystic kidney disease, Meckel–Gruber syndrome, Joubert syndrome, or Bardet–Biedl and Alström syndrome, the last two characterized also by disorders of energy metabolism, have been linked to primary cilia dysfunction [16,17]. A study using cell lines and mouse models demonstrated that the MC4R protein, a member of the melanocortin receptor family and a well-known regulator of energy homeostasis and food intake, is localized in cilia [18]. Functional primary cilia play a requisite role in pancreatic endocrine cell differentiation, morphology, and function [19]. Primary cilia are sensors of the islet environment. Therefore, they are very sensitive to small changes in the local islet microenvironment. Dysfunction in this sensory mechanism may reduce the ability of the beta cells to adapt to these changes. The primary cilia can detect both acute changes, which could provide feedback to dial up or down hormone secretion, and more sustained changes, which could be used for transcriptional reprogramming and long-term adaptation [20]. The cilia can not only detect signals but also transmit information. They mediate trafficking of insulin receptors to the cell surface [21]. Two studies have reported that human pancreatic islet cells express motile cilia genes and proteins and that these motile components mediate active movement of primary cilia via ATP hydrolysis, a key energy-generating reaction [22,23]. Beta cell cilia movement occurs in response to extracellular glucose and is coupled to glucose-stimulated Ca^2+^ influx into beta cells and insulin secretion. According to a study by Walker et al., disruptions in these motile cilia gene regulatory modules are associated with early-stage beta cell-intrinsic defects in T2DM.

Our study in rats showed that knock-out *Nme7−/−* animals lacking completely the *Nme7* gene displayed a variety of symptoms consistent with the presentation of primary ciliary dyskinesia [24]. Heterozygous *Nme7+/−* rats had elevated insulin levels along with decreased glucose tolerance. In addition, these animals showed fibrosis of pancreatic islets [25]. Regarding human studies, the first results showing the possible involvement of *NME7* genetic variability in glucose metabolism come from research conducted on the French-Canadian population from the Saguenay-Lac-St-Jean region of Quebec (Canada), demonstrating a link of the region on chromosome 1 overlapping *NME7* and *BLZF1* genes to insulin resistance [26].

Thus, considering the association of *Nme7* gene defects in mice with primary ciliary dyskinesia coupled with glucose dysregulation and due to the presumed importance of cilia motility for beta cell function also in humans, we have investigated the *NME7* genetic variability in relation to glucose processing in a large sample of adults with varying degrees of insulin sensitivity.

### 1.4. Aims

Our aim was to evaluate and possibly specify the presumed interconnection between genetic variability in the *NME7* gene and glucose processing in humans. For this purpose, we comprehensively characterized anthropometric and biochemical profiles of a representative cohort of 1262 volunteers showing a wide range of glucose tolerance states, including healthy individuals, people with IFG, and IGT, and also T2DM newly diagnosed by our examination. In all participants, we genotyped 8 intronic single nucleotide polymorphisms (SNPs) in the *NME7* gene and its immediate vicinity.

When inviting volunteers, we focused on people with a higher risk of glucose metabolism disorders (described in more detail in Section 1.5) in order to cover the full range of disorder progression from mild to overt T2DM in sufficient representation and thus increase the probability of finding a connection between the pathology and variability of the gene under investigation.

In biochemical evaluation, we focused on the detailed typology of glycemic curves, which was made possible by the use of an extended 3 h OGTT with 30 min intervals of blood glucose measurements. We also included both established and innovative indices of insulin sensitivity and beta cell function calculated from fasting as well as dynamic OGTT conditions with the aim to specify in as much detail as possible any metabolic differences depending on the given *NME7* genotypes.

### 1.5. Study Subjects

In the years 2001–2023, adult Czech individuals with varying degrees of glucose tolerance were continuously examined at the Institute of Endocrinology in Prague. Examinations were based on genetic, anthropometric, and biochemical characterization, including the 3 h OGTT. A complete characterization was conducted on 1262 individuals aged 18 to 75 years, comprising 1035 women (median age 33.3 years) and 227 men (median age 34.0 years). Details characterizing the participants anthropometrically and biochemically are presented in Table 1 in Section 1.5. To expand the proportion of participants with impaired glycoregulation compared to their representation in the population, we approached direct descendants of type 2 diabetics; these made up 26.0% of our cohort based on questionnaire survey. Furthermore, among women, 454 participants (43.9%) had a positive personal history of gestational diabetes mellitus (GDM), and 189 (18.3%) were diagnosed with polycystic ovary syndrome (PCOS) according to the European Society of Human Reproduction and Embryology consensus, as both of these diagnoses increase the risk of developing glucose homeostasis disorders, so the examination was of great importance to the volunteers also on a personal level. The history of GDM was known thanks to cooperation with the Department of Obstetrics and Gynecology, 1st Faculty of Medicine, Charles University, and General University Hospital in Prague. Regarding the diagnosis of PCOS, the Institute of Endocrinology in Prague has been focusing on this endocrinopathy for a long time, and inviting participants for the study took place in cooperation with our experts.

Exclusion criteria for participation in the study were ages below 18 and above 75 years, pregnancy, overt T2DM, or other diseases where undergoing a glucose test would pose a health risk, for example, migraine conditions. The supervising physician always decided on the eligibility to undergo the test after an initial consultation. The study protocol was conducted according to the guidelines of the Declaration of Helsinki and was approved by the Ethics Committee of the Institute of Endocrinology (EK_EÚ_10062019, approval date 10 June 2019). The privacy rights of all the participants have been observed; they signed an informed consent concerning the course of the examination and had the opportunity to ask questions before participating in the study.

## 2. Results

### 2.1. Metabolic Profile of the Cohort

Based on our examination, 17 participants (12 women, 1.2%, and 5 men, 2.2%; *p* = 0.21) were newly diagnosed with T2DM. Impaired fasting glucose was present in 100 individuals (76 women, 7.4%, and 24 men, 10.8%; *p* = 0.10), and impaired glucose tolerance based on the 120th minute of the OGTT was detected in 83 participants (69 women, 6.7%, and 14 men, 6.3%; *p* = 1.00). Impaired glucose metabolism (IGM), encompassing either IFG, IGT, both disorders concurrently, or newly diagnosed T2DM, was present in 167 participants (131 women, 12.6%, and 36 men, 15.9%; *p* = 0.19). According to the NCEP_ATPIII criteria, 139 participants (102 women, 10.0%, and 37 men, 16.4%; *p* = 0.01) suffered from metabolic syndrome.

Table 1 reports detailed anthropometric data and biochemical characteristics of the examined subjects expressed as medians [95% LCL; 95% UCL]. Details related to anthropometric and metabolic differences between men and women in a given cohort have already been published [14]. Given the focus of the study, special attention was paid to glucose metabolism. Therefore, indices of insulin sensitivity were calculated both for fasting and for dynamic conditions following the glucose load. Pancreatic beta cell function was also characterized for the fasting state and for dynamic conditions. Details on the calculation of insulin sensitivity and beta cell function indices listed in Table 1 are reported in methodological Section 4.1.

### 2.2. Representation of Individual Types of Glycemic Curves

Among the 1262 glycemic curves evaluated, 633 (50%) were monophasic, 221 (17.5%) biphasic, and 351 (28%) triphasic, whereas 57 (4.5%) showed more complex patterns.

People in the monophasic and triphasic groups were on average three years older than people in the biphasic group. BMI was on average 1.3 kg/m^2^ higher in people within the monophasic group compared to the other groups. Data presenting age and BMI as the basic characteristics of the given groups based on the glycemic trajectories are shown in Table 2.

The representation of men and women within individual types of curves was significantly different, with the most noticeable difference in the distribution of the bi- and triphasic curves. Statistical comparison showed that a higher percentage of men had a biphasic curve (33% vs. 14% of women) and a higher percentage of women had a triphasic curve (30% vs. 19% of men), Chi^2^ = 46; *p* < 0.001.

The monophasic shape of the curve was predominant in people with impaired glycoregulation: in newly diagnosed diabetics (15 out of 17, i.e., 88%), in subjects with IFG (70 out of 100, i.e., 70%), and in IGT in the 120th minute of the test (65 out of 83, i.e., 78%). Furthermore, 75% of subjects meeting the criteria for metabolic syndrome also fell into the group with a monophasic course of the curve. The monophasic group also included 53% of women with a history of GDM and 46% of women diagnosed with PCOS. These findings suggest that the monophasic group shows worse metabolic health compared to individuals with biphasic, triphasic, or more complex curves and is associated with the presence of components of the metabolic syndrome. In contrast, the biphasic trajectory is protective in terms of metabolic health, as it is associated with the best glycemic and lipid profile. A graphical representation of the different shapes of glycemic curves for the given cohort, as well as details related to differences in metabolic health depending on the shape of the glycemic curve in a cohort studied, have already been published [14].

### 2.3. Genetic Determination of Glycemic Curve Shapes

**Figure 1:**
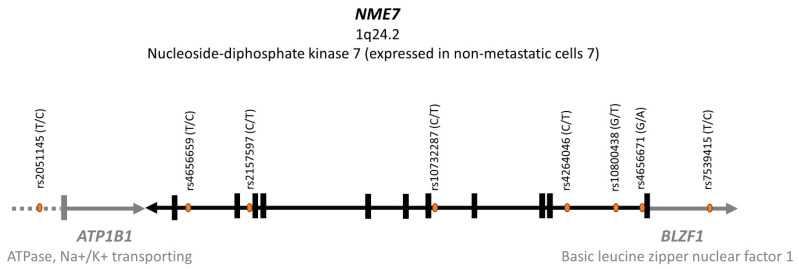
Diagram of the *NME7* gene and its surroundings with indication of the position of the studied SNPs with their major/minor variants in parentheses.

Of the 8 SNPs that were tested in the *NME7* gene and in its immediate vicinity on chromosome 1q24.2 (Figure 1), five variants showed association with the shape of the glycemic curve during the 3 h OGTT. Four of them were significantly associated with the shape of the curves, while a fifth polymorphism showed borderline significance. Table 3 presents the distribution of associated *NME7* genotypes among individual glycemic trajectories. In Supplementary Tables S1–S5, we report detailed Chi^2^ statistical outputs with exact numbers of these five genetic *NME7* variants and the result of the analyses carried out separately for men and women (Supplementary Tables S1–S5).

Proceeding from the arrangement of individual *NME7* SNPs on the chromosome, we found an association of the biphasic type of curves with five tightly linked polymorphisms. More precisely, as shown in Table 3, the wild-type alleles as well as wild-type homozygotes of the rs4656659 (wild-type allele T/homozygotes TT) and of the rs2157597 (wild-type allele C/homozygotes CC) SNPs were less frequent in the biphasic group of curves (*p* = 0.01 and 0.05, respectively). Conversely, the wild-type alleles and wild-type homozygotes of three other linked *NME7* SNPs, rs10732287 (wild-type allele C/homozygotes CC), rs4264046 (wild-type allele C/homozygotes CC), and rs10800438 (wild-type allele G/homozygotes GG), were more frequent in the biphasic group (*p* < 0.01, *p* = 0.01, and *p* = 0.03, respectively).

**Figure 2:**
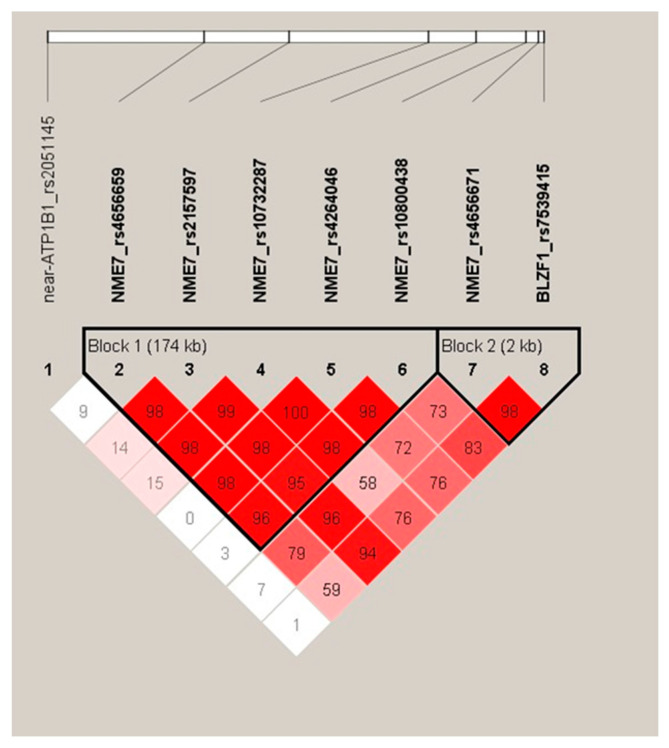
Graph of linkage disequilibrium (LD) between the studied polymorphisms of the *NME7* gene. Shading represents the magnitude of the pairwise LD, with a red-to-white gradient reflecting higher-to-lower LD values.

As demonstrated by the results of linkage analysis (Figure 2), all these five *NME7* variants are in very strong linkage disequilibrium (LD) and form one LD block marked as Block 1. The sixth tested polymorphism of the *NME7* gene, rs4656671, already falls into a different LD block (marked as Block 2) and does not show a significant association with glycemic curve shapes.

### 2.4. Haplotype Analysis

Performed haplotype analysis showed that the two most abundant Block 1 haplotypes, which are inherited in intact formation with very rare recombination events, are precisely the ones indicated by association analysis. The first haplotype forming the TCTTT constellation carries the wild-type variants of SNPs rs4656659 (variant T) and rs2157597 (variant C) in combination with the minor variants of SNPs rs10732287 (variant T), rs4264046 (variant T), and rs10800438 (variant T). It was calculated to be, either in the heterozygous or homozygous formation, highly likely present in 52.2% of participants. The second most abundant haplotype, essentially the inverse of the first, forms the CTCCG constellation and includes the minor variants of SNPs rs4656659 (variant C) and rs2157597 (variant T) in combination with wild-type variants of SNPs rs10732287 (variant C), rs4264046 (variant C), and rs10800438 (variant G). Its presence—again either in heterozygous or homozygous combination—was calculated as most probable in 49.7% of individuals in our cohort. The estimated frequency of other haplotypes with different SNP combinations was already significantly lower, as demonstrated in Table 4, which is a consequence of a very tight genetic link between the polymorphisms in a given haplotype constellation. Three individuals had to be excluded from the haplotype analysis due to insufficient genotyping data.

Distribution of Block 1 haplotypes with a higher than rare representation within individual types of glycemic curves is presented in Table 5. The presence of the most frequent haplotype, TCTTT, was statistically significantly lower in individuals with a biphasic course of the curve compared to individuals with other curve types (Chi^2^ = 15.41, *p* < 0.01), while the presence of the second most frequent haplotype, CTCCG, was significantly higher in individuals with a biphasic course of the curve in comparison with those having different curve types (Chi^2^ = 5.05, *p* = 0.02).

### 2.5. Haplotypes and Biochemical Parameters

Finding that the biphasic course of the glycemic curve is indicative of considerable health benefits in terms of glycoregulation compared to the other curves, especially the monophasic one [14], led us to consider whether a better biochemical profile can also be found in individuals who were assigned to the CTCCG haplotype by haplotype analysis, as this haplotype was shown to be linked with the biphasic course of the glycemic curve. We compared carriers of this haplotype (either in heterozygous or homozygous constellation) with non-carriers, and we focused on glucose metabolism and components of the metabolic syndrome. In connection with the data presented, it is important that the representation of the CTCCG haplotype was the same in both sexes (49.3% in women and 51.8% in men; *p* = 0.51); therefore, the cohort was evaluated as a whole except for parameters with different reference ranges for women and men (WHR, HDL cholesterol). Table 6 demonstrates anthropometric and metabolic characterization of the subjects depending on the presence of the CTCCG haplotype as estimated by haplotype analysis.

Although there were no significant differences between carriers and noncarriers of a given haplotype in anthropometry or blood pressure, when it comes to glucose metabolism, its characteristics clearly indicate that the CTCCG haplotype is reflected in glucose processing and confirm our hypothesis that individuals carrying the haplotype have better glucose metabolism conditions. This is evident not only when it comes to fasting levels of glucose, insulin, and C-peptide, but it is also reflected in dynamic parameters such as AUC_Glycemia_, AUC_Insulin_, and AUC_C-peptide_, and, which is remarkable, all presented indices of insulin sensitivity.

## 3. Discussion

In the present study, we evaluated the association between the course of the glycemic curve during a 3 h OGTT and genetic variability in *NME7*, a gene described as essential for cilia motility of pancreatic beta cells coupled to insulin secretion. According to our observations made on a representative sample of 1262 Czech volunteers, *NME7* variants are associated with the shape of the glycemic curve during the OGTT, a test reflecting the dynamics of glucose processing and thus metabolic health.

Novel results presented here confirm the involvement of the *NME7* gene in human glucose metabolism and demonstrate that common genetic polymorphisms in this gene participate in the individuality of each person’s response to glycemic load. First, there is a strong association of the *NME7* polymorphisms with the shape of the glycemic curve; therefore, the gene is linked to the way the human body processes administered glucose. Given the significant relationship between glycemic curve type and health condition presented both in this and in previously published studies [10,11,12,13,14], it is emerging what potential the projection of *NME7* genetic variability may have on human health. Second, we find it noteworthy to observe that strong linkage disequilibrium between *NME7* variants was clearly detectable already on the basis of OGTT, i.e., on the analysis of glycemic curves. The initial detection of an association of glycemic curves with *NME7* variability, or more precisely the way in which the five variants were associated with the biphasic curve, was subsequently confirmed by performed haplotype analysis. Third, thanks to haplotype analysis and the assignment of individual haplotypes to all subjects, the CTCCG haplotype linked with the biphasic course of the glycemic curve could have been directly tested for the relationship with biochemical parameters. As expected, a connection with glucose metabolism and especially insulin sensitivity was shown, which refers to our initial observation made in the Quebec population [26] that focused our attention on the possible role of the *NME7* gene in glucose metabolism and insulin sensitivity. Also, subsequent studies we conducted on mouse models demonstrated the importance of the gene for maintaining normal glucose tolerance [25]. However, a detailed assessment of the possible clinical impact of *NME7* gene variation is beyond the scope of the current study and remains to be elucidated. It should also be noted that although men had a higher representation of biphasic curves compared to women, as mentioned in Section 2.2, the presentation of the CTCCG haplotype associated with a biphasic curve was the same in both sexes. Therefore, the higher accumulation of men in the group with a biphasic course of the glycemic curve is most likely not mediated by the genetic variability of the *NME7* gene.

In conclusion, our study presents the relationship between the glycemic curve shape and human *NME7* gene variability. Building on previous research linking the gene to glucose processing in rat models, we are now demonstrating for the first time in humans an association of *NME7* haplotypes with the way in which the organism processes the administered glucose. This knowledge is significant, especially in light of the fact that the shape of the glycemic curve during the OGTT is a reflection of different susceptibility to impaired glucose metabolism and other components of metabolic syndrome. Along with proving the link between the shape of the glucose curve and specific haplotypes of the *NME7* gene, we also demonstrate a direct association of a particular haplotype with metabolic health. The fundamental question and the subject of further investigation remain the explanation of the mechanism of how specific intronic variants of the *NME7* gene are reflected in a cascade of biochemical processes affecting the dynamics of glucose homeostasis and related metabolic pathways.

## 4. Materials and Methods

### 4.1. Anthropometric and Metabolic Characterization of the Subjects

Venous blood samples were taken at 8 a.m. after an overnight fast. Glucose metabolism was characterized by blood glucose (enzymatic reference method with hexokinase, Roche, Cobas 6000, Roche Diagnostic, Mannheim, Germany), C-peptide (ECLIA, Roche, Cobas 6000, Roche Diagnostic, Mannheim, Germany), and insulin (ECLIA, Roche, Cobas 6000, Roche Diagnostic, Mannheim, Germany). During the 3 h OGTT (75 g of glucose in 250 mL of water), trajectories of blood glucose, insulin, and C-peptide were analyzed with sampling using a cannula every 30 min of the test. Areas under the glycemic (AUC_Glycemia_), C-peptide (AUC_C-peptide_), and insulin curves (AUC_Insulin_) were calculated. Indices of insulin sensitivity were calculated for the fasting condition (homeostasis model HOMA IR) and for dynamic conditions following the glucose load (ISI_comp_, also known as Matsuda’s index; OGIS calculated for 120 min, MCRest, Si(oral), and PREDIM). Pancreatic beta cell function was characterized by HOMA B for the fasting state and by IGI for dynamic conditions. In addition, the oral disposition index of beta cell function related to insulin sensitivity, IGI × ISI_comp_, was assessed. Also, hepatic extraction was computed by exploiting insulin and C-peptide data through an appropriate formula [27]. Details regarding the calculation methodology of the indices listed in Table 1 and Table 5 have been previously published [13,28,29,30,31].

Lipid profile was assessed by total cholesterol (enzymatic colorimetric test, Roche, Cobas 6000, Roche Diagnostic, Mannheim, Germany), high-density lipoprotein cholesterol (homogeneous enzymatic colorimetric test, Roche, Cobas 6000, Roche Diagnostic, Mannheim, Germany), low-density lipoprotein cholesterol (homogeneous enzymatic colorimetric test, Roche, Cobas 6000, Roche Diagnostic, Mannheim, Germany), and by triacylglycerol concentrations (enzymatic colorimetric test, Roche, Cobas 6000, Roche Diagnostic, Mannheim, Germany).

Thyroid hormones TSH, fT3, and fT4 (ECLIA; Cobas 6000, Roche Diagnostics, Mannheim, Germany) and liver enzymes ALT, AST, and GGT (IFCC method with pyridoxalphosphate; Cobas 6000, Roche Diagnostics, Mannheim, Germany) were evaluated.

Systolic and diastolic blood pressure were measured in a rest state.

Body height and weight were determined to calculate body mass index (BMI), and waist and hip circumferences were measured in order to evaluate body fat distribution and to calculate waist-to-hip ratio (WHR). Metabolic syndrome was diagnosed according to NCEP_ATPIII criteria [32]. Polycystic ovary syndrome was diagnosed based on the European Society of Human Reproduction and Embryology consensus (ESHRE criteria) [33].

### 4.2. Classification of the OGTT Curves

The shape of the glucose curve was assessed according to the methodology of Tura et al. [13]. Glucose trajectory was classified as monophasic if glycemia simply increased and then gradually decreased (one peak). The curve was biphasic if the blood glucose showed a further increase after a previous decrease (two inflection points). The three-phase curve was characterized by two complete peaks. In the 3 h OGTT, much more complex and heterogeneous curve shapes were observed. In some people, there were also four-phase curves with 4 inflections and five-phase curves with 3 complete peaks. These were evaluated together as multiphasic types of glycemic curves.

Variations in shape were considered significant if the difference was at least 2% (this criterion was necessary to avoid the detection of false minima and maxima in the glucose curve), according to the literature focused on this issue [13,34].

### 4.3. Molecular Genetic Analysis

We optimized the genotyping protocol and conducted an analysis of the *NME7* gene and its immediate surroundings, focusing on the following 8 intronic variants: rs4656659, rs2157597, rs10732287, rs4264046, rs10800438, and rs4656671 in *NME7*, rs2051145 near *ATP1B1*, and rs7539415 in *BLZF1.* DNA for genetic analyses was isolated from leukocytes of peripheral blood (QuickGene 610L). Genotyping was carried out through allelic discrimination based on the principle of the real-time PCR method using TaqMan probes (Thermo Fisher Scientific Inc., Carlsbad, CA, USA) on the Light Cycler 480 (Roche, Basel, Switzerland) device. In Supplementary Table S6, TaqMan™ SNP Genotyping Assay IDs are provided for assay specification (Supplementary Table S6). All assays used were functionally tested by the manufacturer.

Linkage analysis was performed using Haploview software (Haploview 4.1, Broad Institute of MIT and Harvard, Cambridge, MA, USA).

Haplotype analysis was then performed using Phase version 2.1.1 [35,36,37], which generated the most likely form of haplotypes for each individual. Haplotype analysis was first processed for all 8 SNPs shown in Figure 1 and Figure 2, including 2 variants in the vicinity of the *NME7* gene, rs7539415 of the *BLZF1* gene upstream, and rs2051145 near the *ATP1B1* gene downstream of the *NME7* locus. This approach led to the generation of 41 distinct haplotype variants, many of which, however, were extremely rare. Therefore, only 5 SNPs forming Block 1 and haplotype variants found in 2.5% or more individuals were kept for statistical evaluation.

### 4.4. Calculations and Statistical Evaluation

Appropriate experimental calculations and data analysis methods for assessing both IS and beta cell function were used [13,27,28,29,30,31]. To evaluate the frequency distribution of individual alleles in the selected genetic polymorphisms, frequency tables were prepared. To assess deviation from the Hardy–Weinberg equilibrium, the Chi^2^ test was applied. Differences in biochemical and anthropometric data were tested by the non-parametric Mann–Whitney test. The Kruskal–Wallis Z-value test with Bonferroni correction was used for multiple comparisons. A two-sided *p*-value of less than 0.05 was assumed to be statistically significant. The power analysis was conducted using NCSS/PASS2020 software (NCSS, LLC. Kaysville, UT, USA).

## Figures and Tables

**Table 1:** Anthropometric and metabolic characterization of the subjects.

Parameter (Units)	Median [95% LCL; 95% UCL]	Reference Limits
number	1262	N/A
age (years)	33.3 [32.6; 34.0]	N/A
systolic blood pressure (mmHg)	115.0 [114.0; 115.0]	100–140
diastolic blood pressure (mmHg)	72.0 [71.0; 73.0]	65–90
BMI (kg/m^2^)	23.9 [23.5; 24.3]	18.5–24.9
WHR women	0.764 [0.759; 0.768]	<0.85
WHR men	0.864 [0.849; 0.877]	<0.95
**glucose metabolism**		
basal glycemia (mmol/L)	4.8 [4.7; 4.8]	3.9–5.5
AUC_Glycemia_(mmol × min/L)	1046 [1031; 1059]	n.s. *
basal insulinemia (mIU/L)	6.16 [5.90; 6.30]	n.s. *
AUC_Insulin_ (pmol × min/L)	33,885 [32,598; 35,091]	n.s. *
basal C-peptide (nmol/L)	0.59 [0.58; 0.60]	n.s. *
AUC_C-peptide_ (pmol × min/L)	3.6 × 10^5^ [3.5 × 10^5^; 3.7 × 10^5^]	n.s. *
hepatic insulin extraction (%)	68.1 [67.5; 68.8]	n.s. *
**insulin sensitivity/resistance**		
HOMA IR	1.30 [1.26; 1.36]	n.s. *
OGIS 120 min (mL/min/m^2^)	457.7 [453.5; 462.1]	n.s. *
ISI_COMP_ ([(mg/dl)^2^(μU/)mL^2^]^−1/2^)	8.48 [8.18; 8.81]	n.s. *
MCRest (mL/min/kg)	9.87 [9.75; 9.99]	n.s. *
Si_(oral)_ ((mL/min/kg)/(μU/)mL)	0.15 [0.15; 0.16]	n.s. *
PREDIM (mg/min/kg)	6.97 [6.83; 7.17]	n.s. *
**beta cell function**		
HOMA B (mIU/mmol)	103.5 [98.8; 106.2]	n.s. *
IGI (pmol/mmol)	88.5 [84.2; 95.1]	n.s. *
Ins_0_/Glc_0_ (pmol/mmol)	7.68 [7.40; 8.00]	n.s. *
AUC_Insulin_/AUC_Glc_ (pmol/mmol)	32.7 [31.9; 33.9]	n.s. *
IGI × ISI_COMP_	264.9 [257.9; 270.2]	n.s. *
**lipid spectrum**		
total cholesterol (mmol/L)	4.59 [4.54; 4.65]	2.9–5.0
HDL cholesterol in women (mmol/L)	1.57 [1.54; 1.60]	1.2–2.7
HDL cholesterol in men (mmol/L)	1.26 [1.21; 1.32]	1.0–2.1
LDL cholesterol (mmol/L)	2.56 [2.52; 2.63]	1.2–3.0
TAG (mmol/L)	0.86 [0.82; 0.89]	0.45–1.70
**liver enzymes**		
ALT (ukat/L)	0.30 [0.30; 0.31]	0.17–0.58
AST (ukat/L)	0.35 [0.34; 0.36]	0.17–0.60
GGT (ukat/L)	0.23 [0.22; 0.24]	0.10–0.70
**thyroid hormones**		
TSH (mIU/L)	2.25 [2.17; 2.33]	0.27–4.20
fT3 (pmol/L)	4.90 [4.85; 4.97]	3.10–6.80
fT4 (pmol/L)	15.2 [15.0; 15.3]	12.0–22.0

Data are given as median [95% LCL; 95% UCL]. BMI—body mass index; WHR—waist-hip ratio; AUCGlycemia—area under the glycemic curve; AUCInsulin—area under the insulinemic curve; AUCC-peptide—area under the C-peptide curve; HOMA IR—Homeostatic Model Assessment of Insulin Resistance; OGIS—Oral Glucose Insulin Sensitivity; ISICOMP—Insulin Sensitivity Composite index; PREDIM—Peripheral Insulin Sensitivity Index; HOMA B—Homeostatic Model Assessment of beta cell function; IGI—Insulinogenic index; IGI × ISICOMP—product of the IGI and the ISICOMP index; HDL—high-density lipoprotein; LDL—low-density lipoprotein; TAG—triacylglycerols; ALT—alanine aminotransferase; AST—aspartate aminotransferase; GGT—γ-glutamyl transferase; TSH—thyrotropin; fT3—free triiodothyronine; fT4—free thyroxine. * n.s.—reference limits are not specified for the given parameter.

**Table 2:** Age and BMI of the groups based on the shape of glycemic curves.

n = 1262	Monophasic n = 633	Biphasic n = 221	Triphasic n = 351	Multiphasic n = 57	*p*-Level
age (years)	34.1 [33.2; 35.1]	31.4 [28.3; 32.9]	34.0 [32.4; 34.7]	28.7 [26.6; 30.6]	**<0.01**
BMI (kg/m^2^)	24.6 [24.1; 25.1]	23.3 [22.6; 24.1]	23.2 [22.7; 23.8]	23.5 [22.2; 24.2]	**<0.01**

Data are given as median [95% LCL; 95% UCL], *p*-level according to Kruskal–Wallis Z-value test, and significant differences are in bold.

**Table 3:** Genotype and allele frequencies of *NME7* gene polymorphisms in individual groups of glycemic trajectories.

***NME7*_rs4656659**	GENOTYPES (%)			ALLELES (%)		
	**TT**	**CT**	**CC**	**T**	**C**	STAT_Genotype distribution:_
monophasic (n = 632)	45	43	12	67	33	Chi^2^ = 16.43 Power = 0.88 ***p*-level = 0.012**
biphasic (n = 219)	34	54	12	61	39	
triphasic (n = 351)	39	53	8	66	34	
multiphasic (n = 56)	48	40	12	68	32	
***NME7*_rs2157597**	GENOTYPES (%)			ALLELES (%)		
	**CC**	**CT**	**TT**	**C**	**T**	STAT_Genotype distribution:_
monophasic (n = 631)	52	40	8	71	29	Chi^2^ = 12.64 Power = 0.76 ***p*-level = 0.049**
biphasic (n = 219)	42	48	10	66	34	
triphasic (n = 350)	48	47	5	72	28	
multiphasic (n = 57)	52	41	7	73	27	
***NME7*_rs10732287**	GENOTYPES (%)			ALLELES (%)		
	**CC**	**CT**	**TT**	**C**	**T**	STAT_Genotype distribution:_
monophasic (n = 632)	46	43	11	67	33	Chi^2^ = 19.39 Power = 0.93 ***p*-level = 0.003**
biphasic (n = 219)	59	33	8	76	24	
triphasic (n = 351)	43	48	9	67	33	
multiphasic (n = 57)	37	48	15	61	39	
***NME7*_rs4264046**	GENOTYPES (%)			ALLELES (%)		
	**CC**	**CT**	**TT**	**C**	**T**	STAT_Genotype distribution:_
monophasic (n = 628)	29	49	22	53	47	Chi^2^ = 16.42 Power = 0.88 ***p*-level = 0.012**
biphasic (n = 217)	39	44	17	61	39	
triphasic (n = 348)	28	56	16	56	44	
multiphasic (n = 57)	25	53	22	52	48	
***NME7***_**rs10800438**	GENOTYPES (%)			ALLELES (%)		
	**GG**	**GT**	**TT**	**G**	**T**	STAT_Genotype distribution:_
monophasic (n = 631)	31	49	20	56	44	Chi^2^ = 14.21 Power = 0.82 ***p*-level = 0.027**
biphasic (n = 219)	42	43	15	64	36	
triphasic (n = 351)	32	53	15	59	41	
multiphasic (n = 56)	24	57	19	53	47	

Data are given as percentages, *p*-level according to Chi^2^ test, and significant differences are in bold.

**Table 4:** Block 1 haplotypes with a higher than rare representation (>2.5%) sorted by frequency.

Haplotype	Number of Carriers		
	Women (n = 1033)	Men (n = 226)	All (n = 1259)
1. TCTTT	550 (53.2%)	107 (47.3%)	657 (52.2%)
2. CTCCG	509 (49.3%)	117 (51.8%)	626 (49.7%)
3. TCCCG	377 (36.5%)	91 (40.3%)	468 (37.2%)
4. TCCTT	209 (20.2%)	47 (20.8%)	256 (20.3%)
5. CCCCG	110 (10.6%)	29 (12.8%)	139 (11.0%)
6. TCCTG	54 (5.2%)	11 (4.9%)	65 (5.2%)

The individuals carried at least one copy of a given haplotype; some may carry two identical copies of the same haplotype.

**Table 5:** Distribution of non-rare Block 1 haplotypes within the types of glycemic curves.

Curve Type	Number of Carriers					
	1. TCTTT	2. CTCCG	3. TCCCG	4. TCCTT	5. CCCCG	6.TCCTG
monophasic	339 (54%)	298 (47%)	224 (35%)	137 (22%)	63 (10%)	33 (5%)
biphasic	88 (40%)	124 (57%)	87 (40%)	50 (23%)	23 (11%)	14 (6%)
triphasic	197 (56%)	179 (51%)	134 (38%)	59 (17%)	47 (13%)	18 (5%)
multiphasic	33 (58%)	25 (44%)	23 (40%)	10 (18%)	6 (11%)	0 (0%)

The individuals included carry at least one copy of a given haplotype; some may carry two identical copies of the same haplotype.

**Table 6:** Anthropometric and metabolic characterization of the subjects according to the presence of the CTCCG haplotype as estimated by haplotype analysis.

Parameter * (Units)	CTCCG Haplotype +	CTCCG Haplotype −	*p*-Level
number	626	633	n/a
age (years)	33.2 [32.3; 34.3]	33.5 [32.4; 34.1]	0.58
systolic blood pressure (mmHg)	114 [113; 115]	115 [114; 116]	0.65
diastolic blood pressure (mmHg)	72 [71; 73]	72 [71; 74]	0.78
BMI (kg/m^2^)	23.7 [23.2; 24.1]	24.2 [23.6; 24.5]	0.10
WHR women	0.760 [0.751; 0.765]	0.769 [0.760; 0.777]	0.11
WHR men	0.862 [0.845; 0.877]	0.867 [0.848; 0.888]	0.49
**glucose metabolism**			
basal glycemia (mmol/L)	4.7 [4.7; 4.8]	4.8 [4.8; 4.8]	**0.01**
AUC_Glycemia_(mmol × min/L)	1029 [1008; 1052]	1059 [1041; 1079]	**<0.01**
basal insulinemia (mIU/L)	5.9 [5.5; 6.2]	6.3 [6.0; 6.7]	**0.04**
AUC_Insulin_ (pmol × min/L)	31,757 [30,510; 33,309]	35,793 [34,209; 38,421]	**<0.01**
basal C-peptide (nmol/L)	0.57 [0.56; 0.59]	0.61 [0.59; 0.63]	**<0.01**
AUC_C-peptide_ (pmol × min/L)	3.5 × 10^5^ [3.4 × 10^5^; 3.6 × 10^5^]	3.7 × 10^5^ [3.6 × 10^5^; 3.8 × 10^5^]	**<0.01**
hepatic insulin extraction (%)	68.5 [67.7; 69.4]	67.7 [66.8; 68.7]	0.14
**insulin sensitivity/resistance**			
HOMA IR	1.24 [1.17; 1.32]	1.37 [1.29; 1.46]	**0.02**
OGIS 120 min (ml/min/m^2^)	462.5 [455.8; 468.6]	453.0 [448.0; 459.3]	**<0.01**
ISI_COMP_ ([(mg/dl)^2^(μU/)mL^2^]^−1/2^)	8.91 [8.56; 9.24]	7.98 [7.52; 8.38]	**<0.01**
MCRest (mL/min/kg)	9.99 [9.81; 10.17]	9.76 [9.54; 9.93]	**<0.01**
Si_(oral)_ ((mL/min/kg)/(μU/)mL)	0.16 [0.15; 0.17]	0.15 [0.14; 0.16]	**<0.01**
PREDIM (mg/min/kg)	7.18 [6.90; 7.38]	6.83 [6.64; 7.00]	**0.03**
**beta cell function**			
HOMA B (mIU/mmol)	103.7 [95.7; 108.3]	103.2 [97.8; 107.3]	0.78
IGI (pmol/mmol)	89.1 [83.1; 95.8]	88.3 [83.2; 98.3]	0.59
Ins_0_/Glc_0_ (pmol/mmol)	7.40 [7.00; 7.85]	7.92 [7.56; 8.37]	0.11
AUC_Insulin_/AUC_Glc_ (pmol/mmol)	31.3 [29.9; 32.7]	34.5 [32.7; 35.8]	**<0.01**
IGI × ISI_COMP_	267.9 [257.7; 275.5]	260.6 [253.3; 269.9]	0.21
**lipid spectrum**			
total cholesterol (mmol/L)	4.61 [4.55; 4.70]	4.58 [4.47; 4.65]	0.54
HDL chol. in women (mmol/L)	1.58 [1.56; 1.64]	1.54 [1.50; 1.59]	**0.03**
HDL chol. in men (mmol/L)	1.30 [1.23; 1.35]	1.21 [1.15; 1.31]	0.28
LDL cholesterol (mmol/L)	2.57 [2.47; 2.64]	2.56 [2.52; 2.64]	0.35
TAG (mmol/L)	0.84 [0.79; 0.88]	0.89 [0.83; 0.93]	0.09
**liver enzymes**			
ALT (ukat/L)	0.30 [0.29; 0.31]	0.30 [0.29; 0.31]	0.59
AST (ukat/L)	0.36 [0.35; 0.37]	0.34 [0.34; 0.35]	0.07
GGT (ukat/L)	0.23 [0.22; 0.25]	0.23 [0.22; 0.24]	0.65
**thyroid hormones**			
TSH (mIU/L)	2.19 [2.11; 2.33]	2.26 [2.19; 2.38]	0.99
fT3 (pmol/L)	4.89 [4.80; 4.99]	4.91 [4.84; 5.00]	0.71
fT4 (pmol/L)	15.0 [14.8; 15.2]	15.4 [15.1; 15.6]	0.13

Data are given as median [95% LCL; 95% UCL], *p*-level according to the Mann–Whitney test, and significant differences are in bold. BMI—body mass index; WHR—waist-hip ratio; AUC_Glycemia_—area under the glycemic curve; AUC_Insulin_—area under the insulinemic curve; AUC_C-peptide_—area under the C-peptide curve; HOMA IR—Homeostatic Model Assessment of Insulin Resistance; OGIS—Oral Glucose Insulin Sensitivity; ISI_COMP_—Insulin Sensitivity Composite index; PREDIM—Peripheral Insulin Sensitivity Index; HOMA B—Homeostatic Model Assessment of beta cell function; IGI—Insulinogenic index; IGI×ISI_COMP_—product of the IGI and the ISI_COMP_ index; HDL—high-density lipoprotein; LDL—low-density lipoprotein; TAG—triacylglycerols; ALT—alanine aminotransferase; AST—aspartate aminotransferase; GGT—γ-glutamyl transferase; TSH—thyrotropin; fT3—free triiodothyronine; fT4—free thyroxine. * Reference limits for the given parameters are stated in Table 1.

## Data Availability

Original raw genotype data processed in the study are available in the public repository Figshare.com, available at https://figshare.com. DOI for public link: 10.6084/m9.figshare.29940992.

## Supplementary Materials

The following supporting information can be downloaded at: https://www.mdpi.com/article/10.3390/ijms26199821/s1.

## Abbreviations

ALT	alanine aminotransferase
AST	aspartate aminotransferase
ATP1B1	sodium/potassium-transporting ATPase subunit beta-1
ATP	adenosine triphosphate
AUC_C-peptide_	area under the C-peptide curve
AUC_Glycemia_	area under the glycemic curve
AUC_Insulin_	area under the insulinemic curve
BLZF1	basic leucine zipper nuclear factor 1
BMI	body mass index
df	degrees of freedom
DNA	deoxyribonucleic acid
fT3	free triiodothyronine
fT4	free thyroxine
GDM	gestational diabetes mellitus
GGT	γ-glutamyl transferase
HDL	high-density lipoprotein
HOMA B	homeostatic model assessment of beta cell function
HOMA IR	homeostatic model assessment of insulin resistance
IFG	impaired fasting glucose
IGI	insulinogenic index
IGT	impaired glucose tolerance
IR	insulin resistance
ISI_COMP_	insulin sensitivity composite index
LCL	lower confidence limit
LD	linkage disequilibrium
LDL	low-density lipoprotein
NME7	non-metastatic cells 7
OGIS	oral glucose insulin sensitivity index
OGTT	oral glucose tolerance test
PCOS	polycystic ovary syndrome
PREDIM	peripheral insulin sensitivity index
SNP	single nucleotide polymorphism
T2DM	type 2 diabetes mellitus
TAG	triacylglycerols
TSH	thyrotropin
UCL	upper confidence limit
WHR	waist-hip ratio
