# Supplementary material for: The NME7 Gene Is Involved in the Kinetics of Glucose Processing
Source: Int J Mol Sci. 2025 Oct 9;26(19):9821. doi: 10.3390/ijms26199821 (PMC12524336; doi:10.3390/ijms26199821)
Supplement: Supplementary file 1 [file ijms-26-09821-s001.zip › ijms-3887144-supplementary.pdf]

International Journal of *Molecular Sciences*

MDPI

Supplementary Materials

Supplementary Table S1-S5 provide exact numbers of individual genotypes of the *NME7* Block 1 SNPs for given glycemic trajectories both in men and women as well as in all participants together.

Supplementary Table S1 for SNP rs4656659

	rs4656659_CT	rs4656659_CC	rs4656659_TT		rs4656659_CT	rs4656659_CC	rs4656659_TT	
**men:**	**observed**			**n**	**expected**			**statistics**
monophasic	47	11	42	100	56,03	8,11	36,01	
biphasic	47	4	22	73	40,88	5,84	26,28	X^2 = 7,904
triphasic	28	2	13	43	24,08	3,44	15,48	df = 6
multiphasic	4	1	4	9	5,04	0,72	3,24	P-level = 0,245
sum	126	18	81	225				
**women:**	**observed**			**n**	**expected**			**statistics**
monophasic	226	64	242	532	243,59	60,77	227,63	
biphasic	70	22	54	146	66,85	16,67	62,47	X^2 = 11,682
triphasic	159	26	123	308	141,03	35,18	131,78	df = 6
multiphasic	18	6	23	47	21,52	5,36	20,11	P-level = 0,069
sum	473	118	442	1033				
**all:**	**observed**			**n**	**expected**			**statistics**
monophasic	273	75	284	632	300,92	68,32	262,74	
biphasic	117	26	76	219	104,27	23,67	91,04	X^2 = 16,432
triphasic	187	28	136	351	167,12	37,94	145,92	df = 6
multiphasic	22	7	27	56	26,66	6,05	23,28	**P-level = 0,012**
sum	599	136	523	1258				

P-level according to Chi^2 test; significant differences are in bold, df—degrees of freedom

Supplementary Table S2 for SNP rs2157597

	**rs2157597_TT**	**rs2157597_CT**	**rs2157597_CC**		**rs2157597_TT**	**rs2157597_CT**	**rs2157597_CC**		
**men:**		**observed**		**n**		**expected**		**statistics**	
monophasic	7	41	52	100	5,30	48,23	46,46		
biphasic	3	40	30	73	3,87	35,20	33,91	X^2	= 5,596
triphasic	1	24	18	43	2,28	20,73	19,97	df	= 6
multiphasic	1	4	5	10	0,53	4,823	4,64	P-level	= 0,469
sum	12	109	105	226					
**women:**		**observed**		**n**		**expected**		**statistics**	
monophasic	47	211	273	531	43,26	224,55	263,18		
biphasic	18	66	62	146	11,89	61,74	72,36	X^2	= 10,814
triphasic	16	140	151	307	25,01	129,82	152,16	df	= 6
multiphasic	3	19	25	47	3,82	19,87	23,29	P-level	= 0,094
sum	84	436	511	1031					
**all:**		**observed**		**n**		**expected**		**statistics**	
monophasic	54	252	325	631	48,19	273,58	309,22		
biphasic	21	106	92	219	16,72	94,95	107,32	X^2	= 12,641
triphasic	17	164	169	350	26,73	151,75	171,51	df	= 6
multiphasic	4	23	30	57	4,35	24,71	27,93	**P-level**	**= 0,049**
sum	96	545	616	1257					

P-level according to Chi^2 test; significant differences are in bold, df—degrees of freedom

Supplementary Table S3 for SNP rs10732287

	**rs10732287_TT**	**rs10732287_CT**	**rs10732287_CC**		**rs10732287_TT**	**rs10732287_CT**	**rs10732287_CC**		
**men:**	**observed**			**n**	**expected**			**statistics**	
monophasic	9	42	49	100	7,522	40,26	52,21		
biphasic	5	21	47	73	5,49	29,39	38,11	X^2	= 13,178
triphasic	1	22	20	43	3,23	17,31	22,45	df	= 6
multiphasic	2	6	2	10	0,75	4,02	5,22	**P-level**	**= 0,040**
sum	17	91	118	226					
**women:**	**observed**			**n**	**expected**			**statistics**	
monophasic	58	233	241	532	55,10	230,72	246,17		
biphasic	12	51	83	146	15,12	63,31	67,55	X^2	= 9,371
triphasic	30	144	134	308	31,90	133,57	142,52	df	= 6
multiphasic	7	20	20	47	4,86	20,38	21,74	P-level	= 0,153
sum	107	448	478	1033					
**all:**	**observed**			**n**	**expected**			**statistics**	
monophasic	67	275	290	632	62,24	270,57	299,18		
biphasic	17	72	130	219	21,56	93,75	103,67	X^2	= 19,392
triphasic	31	166	154	351	34,57	150,26	166,16	df	= 6
multiphasic	9	26	22	57	5,61	24,40	26,98	**P-level**	**= 0,003**
sum	124	539	596	1259					

P-level according to Chi^2 test; significant differences are in bold, df—degrees of freedom

Supplementary Table S4 for SNP rs4264046

	rs4264046_TT	rs4264046_CT	rs4264046_CC		rs4264046_TT	rs4264046_CT	rs4264046_CC		
**men:**		**observed**		**n**		**expected**		**statistics**	
monophasic	20	42	36	98	17,50	46,81	33,68		
biphasic	10	35	28	73	13,03	34,87	25,09	X^2	= 6,088
triphasic	7	24	12	43	7,67	20,54	14,78	df	= 6
multiphasic	3	6	1	10	1,78	4,77	3,43	P-level	= 0,413
sum	40	107	77	224					
**women:**		**observed**		**n**		**expected**		**statistics**	
monophasic	119	268	143	530	105,89	269,13	154,97		
biphasic	27	60	57	144	28,77	73,12	42,10	X^2	= 14,289
triphasic	49	170	86	305	60,94	154,87	89,18	df	= 6
multiphasic	10	23	14	47	9,39	23,86	13,74	**P-level**	**= 0,026**
sum	205	521	300	1026					
**all:**		**observed**		**n**		**expected**		**statistics**	
monophasic	139	310	179	628	123,08	315,50	189,40		
biphasic	37	95	85	217	42,53	109,02	65,44	X^2	= 16,418
triphasic	56	194	98	348	68,20	174,83	104,95	df	= 6
multiphasic	13	29	15	57	11,17	28,63	17,19	**P-level**	**= 0,012**
sum	245	628	377	1250					

P-level according to Chi^2 test; significant differences are in bold, df—degrees of freedom

Supplementary Table S5 for SNP rs10800438

	**rs10800438_TT**	**rs10800438_GT**	**rs10800438_GG**		**rs10800438_TT**	**rs10800438_GT**	**rs10800438_GG**		
**men:**	**observed**			**n**	**expected**			**statistics**	
monophasic	22	39	39	100	16,81	44,69	38,49		
biphasic	7	34	32	73	12,27	32,62	28,10	X^2	= 9,117
triphasic	7	21	15	43	7,23	19,21	16,55	df	= 6
multiphasic	2	7	1	10	1,68	4,46	3,84	P-level	= 0,167
sum	38	101	87	226					
**women:**	**observed**			**n**	**expected**			**statistics**	
monophasic	100	274	157	531	92,70	268,33	169,96		
biphasic	25	60	61	146	25,48	73,77	46,73	X^2	= 10,437
triphasic	46	163	99	308	53,77	155,64	98,58	df	= 6
multiphasic	9	24	13	46	8,03	23,24	14,72	P-level	= 0,107
sum	180	521	330	1031					
**all:**	**observed**			**n**	**expected**			**statistics**	
monophasic	122	313	196	631	109,43	312,23	209,32		
biphasic	32	94	93	219	37,98	108,36	72,65	X^2	= 14,211
triphasic	53	184	114	351	60,87	173,68	116,44	df	= 6
multiphasic	11	31	14	56	9,71	27,71	18,57	**P-level**	**= 0,027**
sum	218	622	417	1257					

P-level according to Chi^2 test; significant differences are in bold, df—degrees of freedom

Supplementary Table S6. Intronic variants determined using TaqMan™ SNP Genotyping Assays

SNP	Nearest gene	Major/Minor allele	TaqMan™ SNP Genotyping Assay ID
**rs2051145**	ATP1B1	T/C	C___1264176_30
**rs4656659**	NME7	T/C	C__28016107_10
**rs2157597**	NME7	C/T	C__16141160_20
**rs10732287**	NME7	C/T	C__32141254_20
**rs4264046**	NME7	C/T	C__32141272_10
**rs10800438**	NME7	G/T	C__32141293_10
**rs4656671**	NME7	G/A	C____288901_10
**rs7539415**	BLZF1	T/C	C__11341886_10

Assays were functionally tested by the manufacturer Thermo Fisher Scientific Inc.
